# Network Pharmacology-Based Elucidation of the Hypoglycemic Mechanism of *Grifola frondosa* GF5000 Polysaccharides via GCK modulation in Diabetic Rats

**DOI:** 10.3390/nu17060964

**Published:** 2025-03-10

**Authors:** Chun Xiao, Chunwei Jiao, Longhua Huang, Huiping Hu, Yizhen Xie, Qingping Wu

**Affiliations:** 1 National Health Commission Science and Technology Innovation Platform for Nutrition and Safety of Microbial Food, Guangdong Provincial Key Laboratory of Microbial Safety and Health, State Key Laboratory of Applied Microbiology Southern China, Institute of Microbiology, Guangdong Academy of Sciences, Guangzhou 510070, China; xiaochun960@hotmail.com (C.X.); hlh1030@126.com (L.H.); hhp201@126.com (H.H.); xieyizhen@126.com (Y.X.); 2 Guangdong Yuewei Edible Fungi Technology Co., Ltd., Guangzhou 510663, China; jiao19800205@126.com

**Keywords:** *Grifola frondosa*, polysaccharides, hypoglycemic mechanism, transcriptomics, proteomics, network pharmacology

## Abstract

Background/Objectives: Our lab has previously reported that *Grifola frondosa* (maitake mushroom) GF5000 has antidiabetic potential owing to its ability to improve insulin resistance. This study aimed to gain insight into the system-level hypoglycemic mechanisms of GF5000 using transcriptomics, proteomics, and network pharmacology. This study provides new insights into the hypoglycemic mechanisms of GF5000, identifying key molecular targets involved in mitigating insulin resistance in T2DM. Methods: Liver protein and gene expression in normal control (NC), diabetic control (DC), and GF5000-treated (GF5000) rats were analyzed via iTRAQ and RNA-seq. The relationships between differentially expressed genes (DEGs), differentially expressed proteins (DEPs), and type 2 diabetes (T2DM) disease targets were studied using Metascape and the Cytoscape GeneMANIA plug-in. Results: One hundred and fifty-two DEGs and sixty-two DEPs were identified; twenty DEGs/DEPs exhibited the same trend in mRNA and protein expression levels when comparing the GF5000 vs. DC groups. The Metascape analysis revealed that the T2DM disease targets included four DEGs—*Gck*, *Scd*, *Abcb4*, and *Cyp3a9*—and two DEPs—glucokinase and acetyl-CoA carboxylase 2. A Cytoscape–GeneMANIA analysis of thirteen DEGs/DEPs related to T2DM showed that *Apoa1*/Apolipoprotein A-I, *Gckr*/glucokinase regulatory protein, and *Gck*/glucokinase had the highest connectivity and centrality in the topological network. The qPCR results confirmed that GF5000 increased the mRNA expression of *GCK* in *GCK*-knockdown HepG2 cells. Conclusions: These results provide theoretical evidence for the use of GF5000 as a potential active nutritional ingredient for the prevention and treatment of T2DM. Our findings suggest that GF5000 targets multiple pathways implicated in T2DM, offering a multi-faceted approach to disease management and prevention.

## 1. Introduction

Diabetes is a major health issue, and its prevalence has reached alarming levels worldwide. Currently, more than half a billion people are living with diabetes worldwide. Type 2 diabetes (T2DM) is the most common type of diabetes, accounting for over 90% of all global diabetes cases [1].

The American Diabetes Association recommends that pharmacological agents for the treatment of T2DM include metformin, a sodium–glucose cotransporter 2 inhibitor or glucagon-like peptide 1 receptor, and dipeptidyl peptidase 4. The principal side effects of these pharmacological agents include gastrointestinal intolerance, diarrhea, cardiovascular comorbidities, hypoglycemia risk, and impacts on weight [2].

For centuries, mushrooms have been regarded as a traditional source of natural bioactive compounds and as potential hypoglycemic and antidiabetic components [3]. *Grifola frondosa*, commonly known as hui-shu-hua in China and maitake in Japan, is an edible and medicinal fungus with nutritional and medicinal value.

The polysaccharides in *G. frondosa* are the main components of the cell wall of the fruiting body, which is mainly composed of α-(1-4), β-(1-4), β-(1-6), and β-(1-3) heteropolysaccharides [4]. These compounds exhibit effects including lowering blood sugar and improving insulin resistance [5,6].

The classical hypoglycemic pathway of *G. frondosa* polysaccharides primarily involves the regulation of insulin signaling pathway-related proteins, including insulin receptor (IR), insulin receptor substrate 1 (IRS1), and phosphatidylinositol-3-kinase (PI3K), to improve insulin resistance in the liver, fat, muscles, and other target organs; polysaccharides that are involved in this process include MT-α-glucan [7,8,9], X peptidoglycan [10,11], SX glycoprotein [12,13], and heteropolysaccharide GFP-N [14]. Another important mechanism involves increasing the expression and activity of enzymes related to the glucose metabolism of Akt/protein kinase B(PKB), glycogen synthase kinase 3 (GSK-3), glucose transporter 4 (GLUT4), activated protein kinase-α (AMPK-α), and glucokinase (GCK); polysaccharides that are involved in this process include heteropolysaccharide GFP [15], GFP-W [16], crude polysaccharide GFWE [17], and GFP [18]. In addition, a few studies have shown that *G. frondosa* polysaccharides may provide health benefits by regulating the structure of the gut microbiota and inhibiting proteins related to inflammatory pathways, such as GFP-N [14].

Consistent with the above studies, our previous research showed that *G. frondosa* polysaccharides F2 and F3 reactivated IR and IRS1 to improve insulin resistance in rats with T2DM induced by streptozotocin (STZ) injection combined with a high-fat diet [19]. In addition, we reported that GF5000 has the potential to increase insulin sensitivity, which is related to the reduction in inflammation via the regulation of the gut microbiota composition [20].

Diabetes is a group of metabolic disorders characterized by long-term hyperglycemia; however, current research on the hypoglycemic effects of *G. frondosa* is focused on several targets, making it difficult to comprehensively determine its mechanism of action.

Therefore, in the present study, transcriptomics, proteomics, and network pharmacology profiling techniques were utilized to reveal the hypoglycemic mechanism of GF5000 at the system level. Our results can be applied to the development of new antidiabetic functional foods and health products.

Despite the known antidiabetic potential of *G. frondosa* polysaccharides, a comprehensive understanding of their hypoglycemic mechanism remains elusive. Therefore, this study aims to elucidate the system-level hypoglycemic mechanisms of GF5000 polysaccharides in diabetic rats using an integrative approach combining transcriptomics, proteomics, and network pharmacology.

## 2. Materials and Methods

### 2.1. G. frondosa Fraction GF5000

The dried fruiting bodies of *G. frondosa* W151021 were homogenized into a fine powder. The powder was mixed with distilled water at a ratio of 1:20 (*w*/*v*) and extracted at approximately 80 °C. The mixture was filtered and centrifuged at 5000× *g* for 10 min at 4 °C. The resulting supernatant was concentrated under reduced pressure (not exceeding 60 °C) and ultrafiltrated (Mw = 5000) to yield the GF5000 fraction.

### 2.2. Hypoglycemic Mechanism of GF5000 in Diabetic Rats

In our previous study [20], oral glucose tolerance testing and serum biochemical parameters were used to assess the hypoglycemic effects of treating diabetic rats with GF5000 (112.5 and 675 mg/kg/d, i.g., 16 male SD rats/group) for 48 d. All of the animal procedures complied with the Guide for the Care and Use of Laboratory Animals and were approved by the Ethical Committee of the Guangdong Laboratory Animals Monitoring Institute (Approval ID: IACUC2016019). At the end of the experimental period, the rats were anesthetized and euthanized via cervical decapitation; their livers were quickly separated, removed, weighed, snap-frozen, and stored at −70 °C.

**Figure 1:**
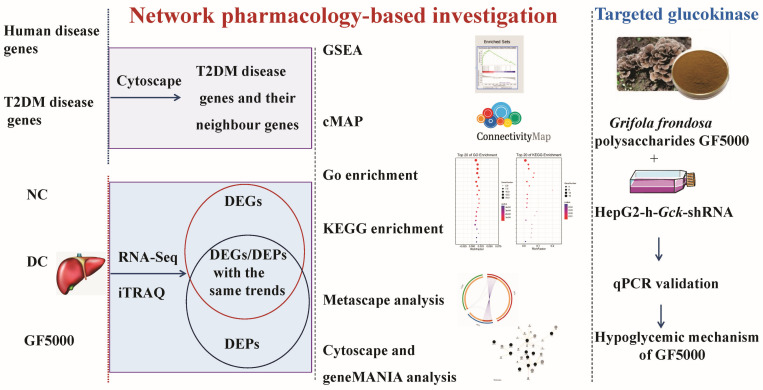
Network pharmacology analysis workflow for transcriptomics and proteomics.

In the present study, the livers of the normal control (NC), diabetic control (DC) (saline), and GF5000-treated (675 mg/kg/d) diabetic groups were analyzed using iTRAQ and RNA-seq. The experiment workflow diagram is illustrated in Figure 1.

### 2.3. RNA-Seq-Based Transcriptomic Analysis

TRIzol reagent (Invitrogen, Carlsbad, CA, USA) was used to isolate the total RNA from each liver sample (three samples/group). A RiboMinus Kit (Invitrogen) was used to remove ribosomal RNA from the total RNA, and the purified RNA was then stored at −80 °C for further use. The total mRNA was cut into 200 bp fragments using lysis buffer, and the first-strand cDNA was synthesized using reverse-transcriptase and random primers.

Subsequently, second-strand cDNA was synthesized using DNA polymerase I and RNase H. The constructed sequencing libraries were sequenced using an Illumina Hiseq 2000 platform (BGI, Shenzhen, China), and raw reads were generated [21]. The correlation value between two samples is very close to 1 when a sample is highly similar to another sample. The correlation values between two samples were calculated based on the FPKM results. The square of the correlation value should be ≥0.92 according to the standard of the Encode plan. After the correlation values of the two biological replicates were calculated, differentially expressed genes (DEGs) between the two groups were selected with a fold change ≥2 and divergence probability ≥0.8 [22]. Genomic sequencing and analysis were conducted by the Beijing Genomics Institute (BGI) according to standard protocols.

### 2.4. ITRAQ-Based Proteomic Analysis

Nine liver tissue samples (80 mg) (three samples/group) were pulverized in liquid nitrogen and sonicated in lysis buffer (8 mol/L urea, 5 mmol/L IAA, 50 mmol/L NH4HCO3, 1× protease cocktail) on ice. As previously mentioned [23], protein concentration was determined using a gel-assisted method.

The extracted protein was hydrolyzed using trypsin and labeled with iTRAQ 8-plex kits. The peptides, labeled with respective isobaric tags, were incubated for 2 h and then fractionated by SCX.

For SCX chromatography using the Shimadzu LC-20AB HPLC Pump system (Kyoto, Japan), iTRAQ-labeled peptide mixtures were reconstituted with 4 mL buffer A (25 mmol/L NaH2PO4 in 25% ACN, pH 2.7) and loaded onto a 4.6 × 250 mm Ultremex SCX column containing 5 μm particles (Phenomenex, Torrance, CA, USA). The peptides were eluted with a gradient of buffer A at a flow rate of 1 mL/min for 10 min, 5–35% buffer B (25 mmol/L NaH2PO4, 1M KCl in 25% ACN, pH 2.7) for 11 min, and 35–80% buffer B for 1 min. The system was then maintained in 80% buffer B for 3 min before equilibrating with buffer A for 10 min before the next injection. Elution was monitored by measuring the absorbance at 214 nm, and fractions were collected every 1 min. The eluted peptides were pooled into 20 fractions, desalted using a Strata X C18 column (Phenomenex), and vacuum-dried [24].

Each fraction was centrifuged at 20,000× *g* for 10 min before the pellet was resuspended in buffer A (2% ACN, 0.1% FA); the average final concentration of peptide was about 0.5 μg/μL. An amount of 10 μL supernatant was loaded onto a LC-20AD nanoHPLC (Shimadzu, Kyoto, Japan), with 2 cm of a C18 trap column. Then, the peptides were eluted onto 10 cm of an analytical C18 column (inner diameter 75 mm). The samples were loaded at 8 μL/min for 4 min, and then the gradient was run at 300 μL/min for 41 min starting from 5 to 35% B (98% ACN, 0.1% FA), followed by a 5 min linear gradient to 80%, maintenance at 80% B for 5 min, and, finally, a return to 5% in 1 min.

Before the tandem mass spectrometry (MS/MS) analysis was conducted using a Q EXACTIVE (Thermo Fisher Scientific, San Jose, CA, USA) coupled to the HPLC online, the peptide was ionized via nano electric spray. Intact peptides were detected at a resolution of 70,000 using Orbitrap.

High-energy collision dissociation (HCD) operation mode was used to select peptides for MS/MS. With the normalized collision energy set to 27.0, ion fragments were detected in the Orbitrap with a resolution of 17,500. In the MS survey scan, a data-dependent program was performed between 1 MS scan and 15 MS/MS scans for the 15 most abundant precursor ions exceeding the threshold ion count of 20,000, followed by a dynamic exclusion duration of 15 S. The applied electrospray voltage was 1.6 kV. Automatic gain control (AGC) was used for optimizing the spectra generated by the Orbitrap. The AGC target for full MS was set to 3E6, and that for MS2 was 1E5. The *m*/*z* scan ranges of 350–2000 and 100–1800 Da were used for the MS and MS2 scans, respectively.

Proteome Discoverer 1.2 (PD 1.2, Thermo) [5600 ms converter] was used to convert the raw MS/MS data into MGF files, which were then searched using Mascot (Matrix Science, London, UK; version 2.3.02) against the rat database (http://www.ncbi.nlm.nih.gov/bioproject/PRJNA169, accessed on 22 June 2017). For protein quantitation, it was required that there were at least two unique peptides from an individual protein. The quantitative protein ratios were weighted and normalized using the median ratio in Mascot. The differentially expressed proteins (DEPs) between two groups were selected as those with fold changes of >1.2 and *p*-values < 0.05. The omics sequencing and analysis were performed according to the BGI standard protocols.

### 2.5. Gene-Set Enrichment Analysis (GSEA)

GSEA was performed to further understand the underlying molecular mechanisms. The reference gene sets (c2.cp.kegg.v7.4. symbols.gmt) were obtained from the Molecular Signatures Database v7.4 (http://software.broadinstitute.org/gsea/msigdb, accessed on 7 September 2021), with the number of permutations set to 1000. Statistically significant gene-set enrichment magnitudes were identified using a normalized enrichment score (NES) > 1, normal *p*-value (NOM *p*-val) < 0.05, and false discovery rate (FDR) < 0.25.

### 2.6. Connectivity MAP (cMAP) Analysis

Upregulated and downregulated transcriptome gene lists were established, and gene ID conversion was carried out on the Affymetrix (https://www.affymetrix.com/site/mainPage.affx, accessed on 3 July 2019) website.

The gene expression profiles associated with GF5000 treatment were input into cMAP (https://www.broadinstitute.org/connectivity-map-cmap, accessed on 24 December 2024) to search for predicted similar small-molecule treatments.

Similarity was represented using a connectivity score between −1 and 1. A high positive connectivity score indicated that the input gene expression profile may be similar to that of a small molecule, while a high negative connectivity score indicated the opposite.

### 2.7. Gene Ontology (GO) and KEGG Pathway Enrichment Analysis of DEGs and DEPs with the Same Trends

GO and KEGG pathway enrichment analyses of DEGs and DEPs with the same expression trends were performed using Omicshare tools (https://www.omicshare.com/, accessed on 24 December 2024).

### 2.8. Metascape Analysis

T2DM drug targets (Table S1) were collected from the Therapeutic Target Database (http://bidd.nus.edu.sg/group/cjttd/, accessed on 12 November 2019) and DrugBank database (https://www.drugbank.ca/, accessed on 12 November 2019).

Metascape (http://metascape.org/) analysis was performed for multiple gene lists of DEGs (152, Rat), DEPs (62, Rat), and T2DM therapeutic target genes (98, Human). A Circos plot was used to display the functional relationships between pairs of genes.

### 2.9. Cytoscape and GeneMANIA Analysis

The human disease gene file (hprdppi.txt) (Table S2), collected from http://www.hprd.org, accessed on 24 December 2024, was input into Cytoscape to construct the background network.

The T2DM drug targets and their neighbor nodes were selected and extracted as a subnetwork (Table S3) containing 831 genes. The rat DEGs/DEPs from the GF5000 group vs. DC group comparison were converted to human DEGs/DEPs.

The genes common to the DEGs/DEPs list (Table S4) and the subnetwork (Table S3) were selected and then analyzed via Cytoscape–GeneMANIA to identify the associated genes, which we will refer to as attention genes (AGs). Finally, the topological networks of the AGs were constructed and analyzed.

### 2.10. Construction of a Stable GCK-Knockdown HepG2 Cell Line with a GCK shRNA Expression Lentiviral System

HepG2 cells were purchased from the Chinese Type Culture Collection (CTCC, China) and grown in 10% FBS/DMEM medium. A lentiviral vector carrying an shRNA targeting the GCK gene and a HepG2 cell line stably expressing GCK shRNA established using this lentiviral system were purchased from Hanbio Biotech (Shanghai, China).

### 2.11. Effect of GF5000 on GCK-Knockdown HepG2 Cells

*GCK*-knockdown HepG2 cells were grown in 10% fetal bovine serum (FBS)/MEM medium or DMEM/F-12 (1:1) medium. Cells were cultured at 37 °C in a 5% humidified CO_2_ atmosphere for 48 h. Then, GF5000 was added to the culture medium for 6 h at a concentration of 20 mg/mL. Cells were harvested by centrifugation and washed twice with ice-cold PBS. 

The total RNA was extracted using an RNA Extraction Kit (Huankai, China), with a DNaseI digestion step. The total RNA concentration was determined by measuring the absorbance at 260 nm. The total RNA was reverse-transcribed using a Plus All-in-one 1st Strand cDNA Synthesis SuperMix (gDNA Purge) Kit (gDNA Purge) (Huankai, China) according to the manufacturer’s instructions. PCR was performed in a real-time thermal cycler using an SYBR qPCR SuperMix Plus kit (Huankai, China) as follows: 40 cycles of denaturation at 95 °C for 5 s and annealing/elongation at 60 °C for 30 s. The primer sequences for *GAPDH* (NM_001256799.3) were 5′-TCAAGGCTGAGAACGGGAAG-3′ and 5′-TCGCCCCACTTGATTTTGGA-3′. The primer sequences for *GCK* (NM_033507.3) were 5′-TCTTAGCCCCTCGGAGAGAT-3′ and 5′-TCTGCTCTACCAGAGTCAAGGC-3′. The SYBR Green assay was used to detect products from the reverse-transcribed cDNA sample. The mRNA signals were normalized to the *GAPDH* mRNA signals in each group. 

### 2.12. Statistical Analysis

The samples underwent three replicate tests, and the obtained data are presented as the mean ± standard deviation (SD). Differences between groups were determined via an analysis using *t*-tests. Statistical differences were considered significant at *p* < 0.05, 0.01, 0.001, and 0.0001 levels. GraphPad Prism software version 10.4 was used for all analyses.

## 3. Results

### 3.1. Overview

**Figure 2:**
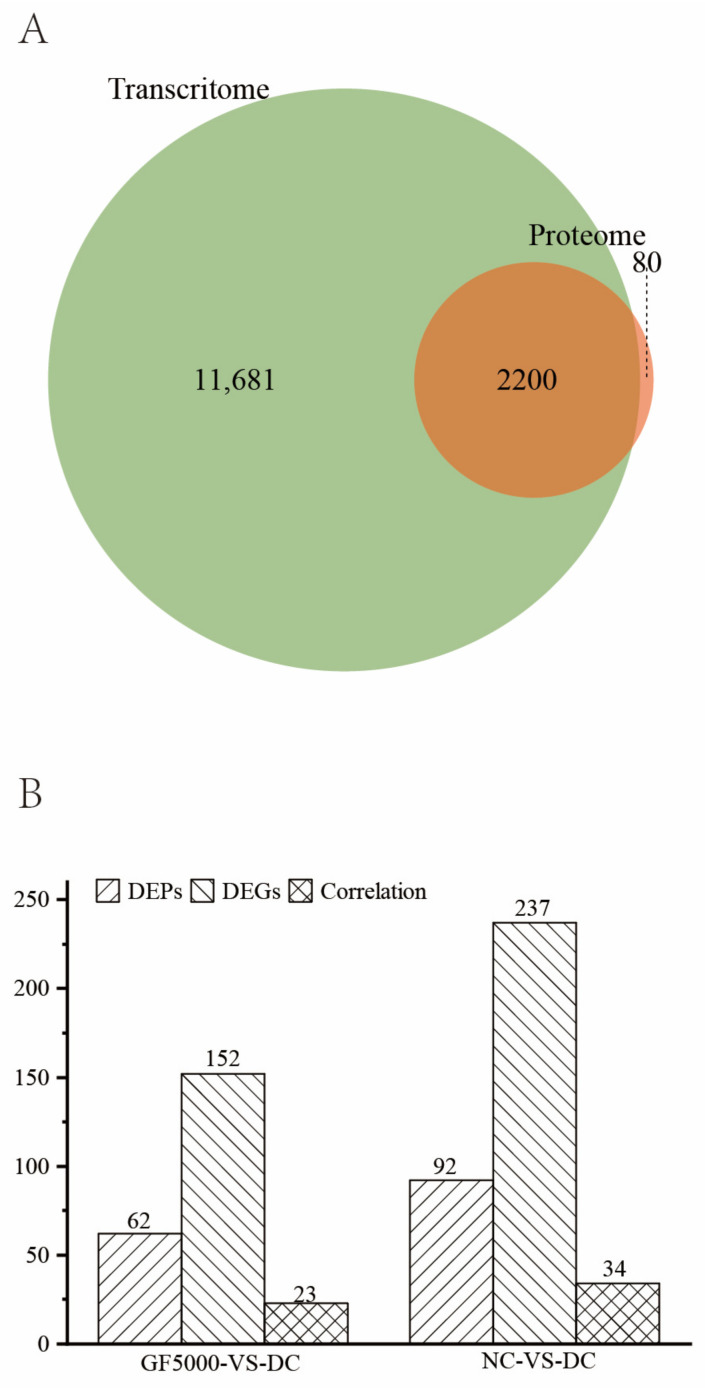
(**A**) Venn diagram of the expressed genes and proteins in all samples. (**B**) Numbers of DEPs and DEGs and the correlation between the proteome and transcriptome in the two comparisons.

We identified 13,881 genes and 2280 proteins in the nine samples; 2200 of the proteins corresponded to transcripts (Figure 2A). There were 62 DEPs and 152 DEGs in the GF5000-VS-DC comparison and 92 DEPs and 237 DEGs in the NC-VS-DC comparison (Figure 2B).

An integrative analysis of the RNA-seq and proteomics data showed that 23 and 34 proteins had corresponding transcripts in the GF5000-VS-DC and NC-VS-DC comparisons, respectively (Figure 2B).

### 3.2. GSEA

**Figure 3:**
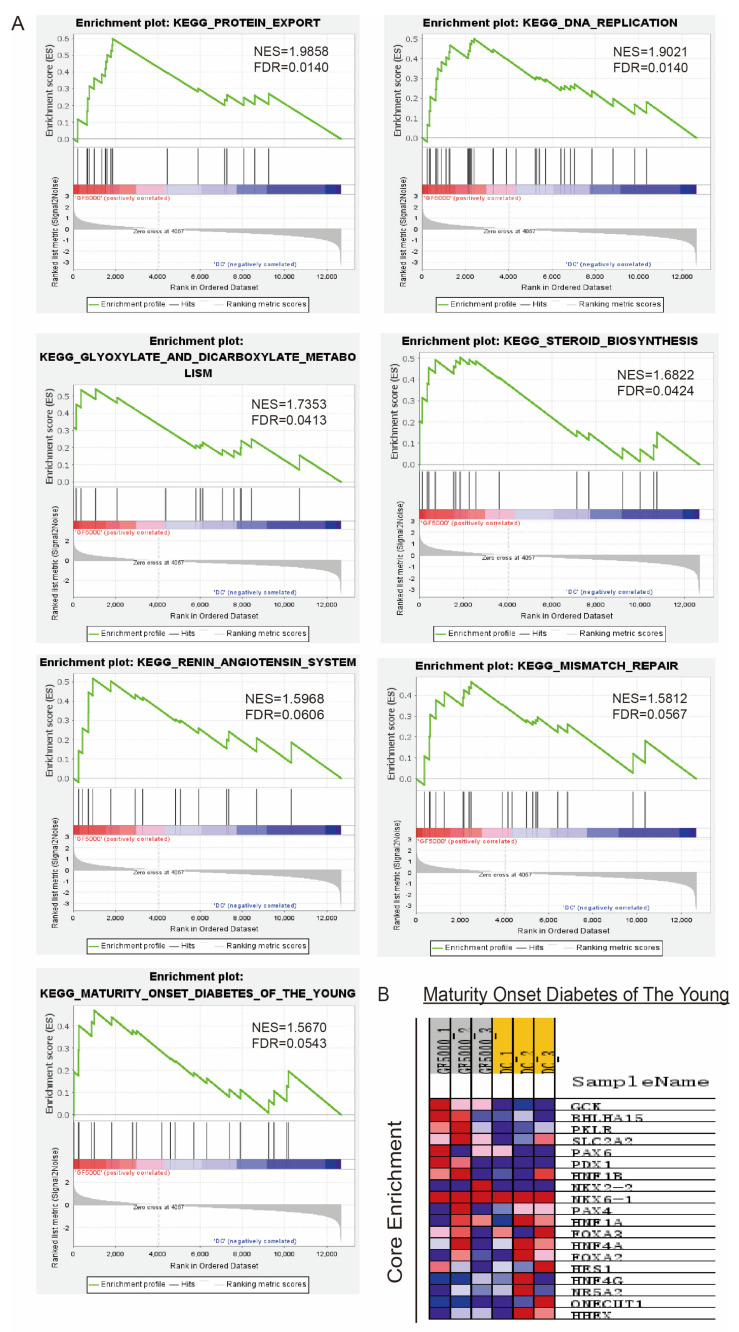
(**A**) Gene-set enrichments in the GSEA, ranked by NES. (**B**) Heat map showing the expression of the core enrichment genes involved in the KEGG pathway of maturity-onset diabetes of the young.

Seventeen gene sets were upregulated in the GF5000 group. Figure 3A shows that eight gene sets were significantly enriched at a nominal *p*-value < 0.05 in the GF5000 group. After GF5000 treatment, the top seven upregulated gene sets according to the normalized enrichment score (NES) had the highest overlapping genes involved in protein export, DNA replication, glyoxylate and dicarboxylate metabolism, steroid biosynthesis, angiotensin system, etc. The expression levels of the core enrichment genes (*GCK*, *BHLHA15*, *PKLR*, *SLC2A2*) involved in maturity-onset diabetes of the young are shown in a heat map in Figure 3B.

### 3.3. Identification of Drugs with Similar Effects Using cMAP Analysis

The top 20 small molecules with a “Score > 0.7” predicted using cMAP are shown in Table 1. There were two common T2DM drug molecules, metformin and rosiglitazone, which had scores of 0.854 and 0.779, respectively.

### 3.4. DEGs and DEPs Showing the Same Trends in the GF5000-VS-DC Comparison

Twenty-three DEGs/DEPs showed the same trends at the transcriptomic and proteomic levels in the GF5000-VS-DC comparison. Among them, there were eleven upregulated DEGs/DEPs (No. 1-11) and twelve downregulated DEGs/DEPs (No. 12-23) (Table 2).

### 3.5. GO and KEGG Pathway Enrichment Analysis of DEGs and DEPs with the Same Trends

**Figure 4:**
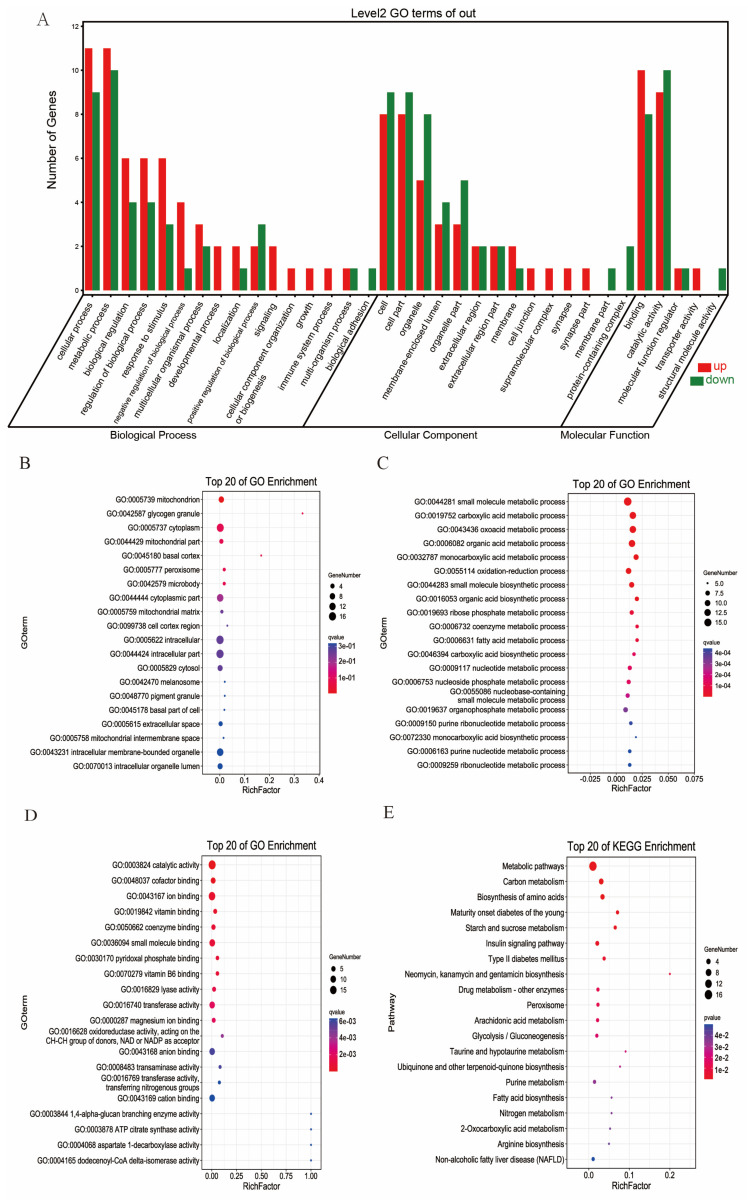
GO terms (**A**); GO enrichment terms as cellular components (**B**), biological processes (**C**), and molecular functions; and (**D**) KEGG pathway enrichment analysis of the twenty-three DEGs/DEPs with the same trends (**E**).

Twenty-three DEGs/DEPs with similar trends were categorized into three main GO categories: 16 biological processes, 5 molecular functions, and 14 cellular components (Figure 4A). Among these subcategories, the GO terms “metabolic process and cellular process”, “binding”, and “cell and cell part” were the most significantly enriched in the biological processes, molecular functions, and cellular components, respectively.

The gene ontology enrichment results for the twenty-three DEGs/DEPs with the same trends are shown in Figure 4B–D, which revealed that these genes may be involved in various processes, including the small-molecule metabolic process, carboxylic acid metabolic process, oxoacid metabolic process, organic acid metabolic process, monocarboxylic acid metabolic process, and oxidation-reduction process; they are primarily located in the mitochondrion and glycogen granule, and their molecular functions are mainly related to catalytic activity and cofactor binding.

The KEGG pathway enrichment analysis of the DEGs and DEPs with the same trends demonstrated that they were potentially involved in metabolic pathways, carbon metabolism, the biosynthesis of amino acids, and maturity-onset diabetes of the young (Figure 4E).

### 3.6. Relationships Among T2DM Disease Genes, DEGs (Human), and DEPs (Human)

**Figure 5:**
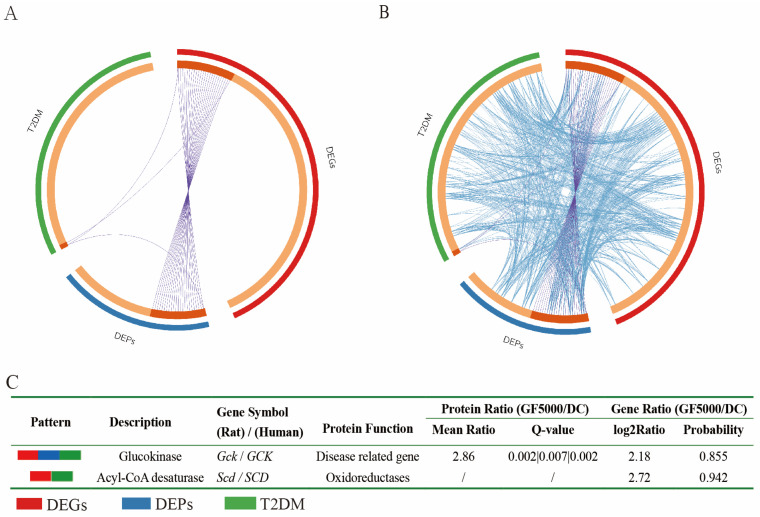
The overlaps between the gene lists (T2DM disease genes, DEGs, and DEPs) are shown in a Circos plot (**A**,**B**). The table of the common genes between DEGs, DEPs, and T2DM disease genes (**C**). The shared genes are linked by purple lines and different genes belonging to the same enriched ontology term are linked by blue lines. The inner circle represents gene lists, where hits are arranged along the arc. Genes that hit multiple lists are colored in dark orange, and genes unique to a list are shown in light orange.

Figure 5A shows the genes shared between DEGs (human), DEPs (human), and T2DM therapeutic targets, and Figure 5B shows the genes from different gene lists that belong to the same enriched ontology terms. There is only one gene (*GCK*) shared between the T2DM disease genes, DEGs, and DEPs and another (*SCD*) shared between the T2DM disease genes and DEGs (Figure 5C).

### 3.7. Analysis of the Genes Common to the Identified DEGs and DEPs in Combination with T2DM Targets

Supplemental Table S3 shows the subnetwork of the T2DM drug targets and their neighbor nodes, which includes 831 genes. There were thirteen common genes drawn from the subnetwork (Table S3) and DEGs/DEPs (Table S4); these are shown in Table 3.

### 3.8. Cytoscape GeneMANIA Network Analysis

**Figure 6:**
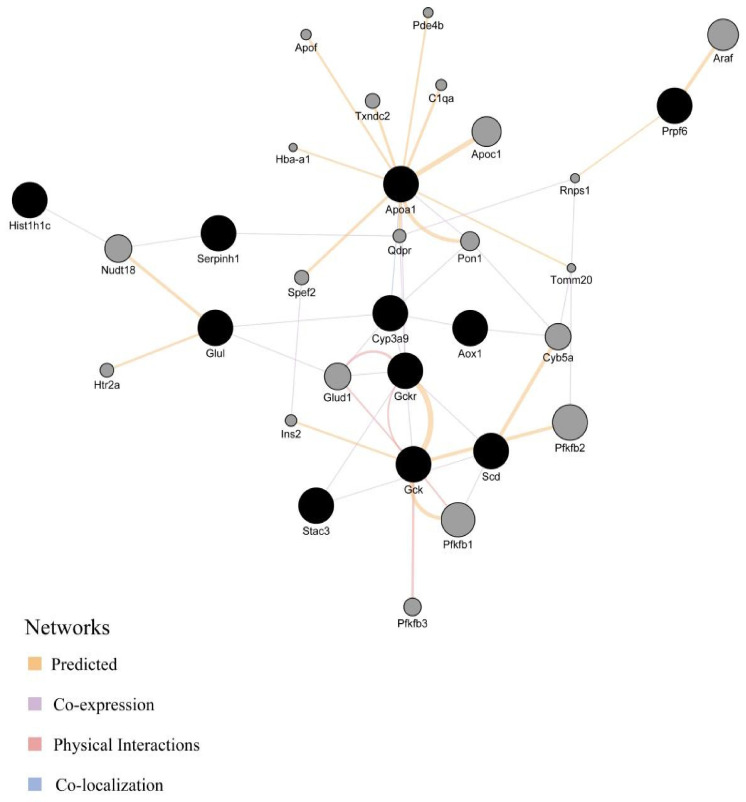
Thirty-one AGs in Cytoscape GeneMANIA network analysis. Black nodes indicate thirteen common genes, gray nodes indicate predicted associated genes, and colored lines indicate different interactions. (Thirteen targets were input into the Cytoscape GeneMANIA analysis platform, but *Abcb4* was not recognized by the system. In the analysis result, *Acaca* was an independent node and was eliminated as it had no connection with other nodes.)

In addition to the thirteen common genes (Table 3), we found twenty genes predicted to be associated with them (Figure 6). Therefore, we actually obtained thirty-one attention genes (AGs) via Cytoscape GeneMANIA network analysis.

Among the interactions of the thirty-one AGs, predicted interactions, co-expression, physical interactions, and colocalization represent 63.97%, 22.02%, 12.81%, and 1.19% of the total interactions, respectively (Table 4).

Among the interactions of the thirty-one AGs, the node with the highest degree of connection is *Apoa1*, followed by *Gckr* and *Gck* (Table 5).

*Apoa1* has thirteen interacting genes, and the predicted functions of these interactions involve co-expression, predicted association, and co-localization. The *Gckr* has ten interacting genes, and the predicted functions of the interactions involve co-expression, physical interactions, and co-localization. *Gck* has nine interacting genes, and the predicted functions of these interactions involve co-expression, physical interactions, and co-localization.

### 3.9. qPCR Analysis of GF5000-Treated GCK-Knockdown HepG2 Cells

**Figure 7:**
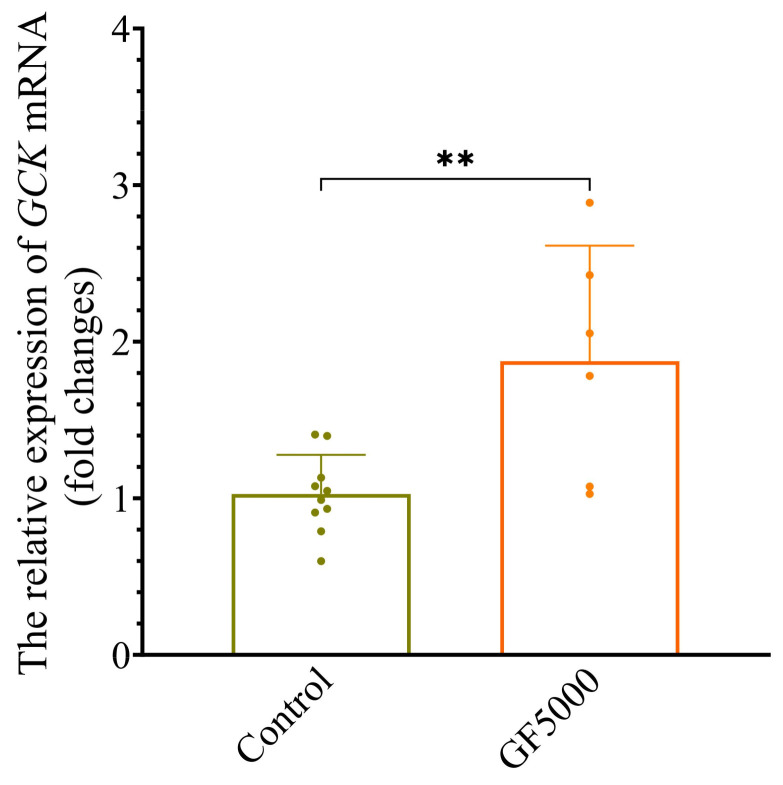
GF5000 increased the mRNA expression of *GCK* (** *p* < 0.01 vs. DC).

Figure 7 shows that the relative expression of *GCK* mRNA in *GCK*-knockdown HepG2 cells increased after treatment with GF5000 (20 mg/mL) for 6 h. Compared with the control group, GF5000 caused an approximately twofold increase in *GCK* mRNA expression (*p* < 0.01).

## 4. Discussion

The cMAP profiles of GF5000-treated diabetic rats were excellent matches with those treated with the diabetes drugs metformin and pioglitazone. Our lab previously reported that GF5000 has the potential to increase insulin sensitivity, which is related to alleviating inflammation by regulating the composition of the gut microbiota [20]. In the present study, transcriptomics, proteomics, and network pharmacology profiling techniques were utilized to reveal the hypoglycemic mechanism of GF5000 at the system level and explore the core targets for lowering blood glucose. The cMAP profiles of GF5000-treated diabetic rats showed an excellent match with diabetes drug of metformin and pioglitazone treatment. Pioglitazone (peroxisome proliferator-activated receptor agonists) and metformin (insulin-sensitizing agent) as antidiabetic drugs in a combined therapy might have a synergistic protective effect on non-alcoholic fatty liver disease (NAFLD) by improving hepatic lipid profiles in HFD-induced mice [25] and newly diagnosed T2DM patients [26], indicating that GF5000 could be a potential drug candidate for T2DM and NALFD.

The KEGG pathway enrichment analysis of the twenty DEGs and DEPs with the same trends demonstrated that they were potentially involved in the carbon metabolism and biosynthesis of amino acids. Carbon metabolism, including one-carbon metabolism and central carbon metabolism, is the main source of energy that provides precursor substrates for acetylation and methylation metabolic reactions. Therefore, it regulates the function of immune cells and influences metabolic diseases, such as T2DM [27,28]. Among the pathways associated with T2DM, amino acid metabolism has been reported to be an important participant in the development of various metabolic diseases, including obesity [29,30]. Over the past few years, many studies have shown that branched-chain amino acids (BCAAs: leucine, valine, and isoleucine) are linked with metabolic markers such as insulin resistance, BMI, TG, and HbA1c [31]. In addition, aromatic amino acids (AAAs: tyrosine, tryptophan, and phenylalanine) and one aliphatic AA (lysine) were found to be associated with the risk of developing T2DM [32]. According to the above, we boldly speculate that the amino acid metabolic pathway enriched by DEGs and DEPs also undergoes changes in the content of BCAAs and AAAs, which is pivotal for the improvement of insulin resistance mediated by GF5000.

In order to explore whether there is a link between differentially expressed genes or proteins and T2DM, Metascape and Cytoscape–GeneMANIA analyses were performed to identify the key genes/proteins *Apoa1*/ApoA-I, *Gck*/GCK, and *Gckr*/GCKR.

One complication of diabetes is lipid metabolism disorder. ApoA-I may play an important role in the GF5000-mediated improvement of lipid metabolism. ApoA-I is the main HDL apolipoprotein and the primary cofactor for the cholesterol-esterifying enzyme, and its levels are decreased in patients with T2DM. Human clinical trials have shown that increased circulating ApoA-I levels improve glycemic control [33]. However, we noticed that the HDL values of the diabetic model were higher than those of the diabetic control group in our previous animal study [19], which was inconsistent with the clinical phenotype. Therefore, although GF5000 reduces ApoA-I protein expression in T2DM rats, we still believe that ApoA-I plays an important role in the regulation of glucolipid metabolism disorders in T2DM rats mediated by GF5000.

GCK, which plays a key role in glucose metabolism, is mainly expressed in liver cells (99%) and pancreatic islet cells (1%). When blood sugar rises after a meal, insulin stimulates GCK to regulate liver glucose absorption and helps to convert postprandial blood sugar into glycogen, which is then stored in the body [34]. Because of its central role in glucose homeostasis, glucokinase has been targeted in drug development, aiming to augment its activity and thereby reduce hyperglycemia in patients with diabetes [35]. Small-molecule stallokinase activators (TTP-355, TDMG-123, and Dorzagliatin) can bind to some amino acid residues (Lys169, Arg63, Tyr215, etc.) of GCK to increase the affinity between the protein and glucose and then activate GCK to lower blood sugar levels [36].

In hepatocytes, GCK is regulated by the GCK regulatory protein (GCKR) and nuclear sequestration at low plasma glucose levels. Following an increase in glucose levels, GCKR is replaced by glucose, the GCK–GCKR complex dissociates, and the levels of GCK in the cytoplasm are markedly increased, thereby leading to an increase in GCK activity [37]. As a natural regulator of GCK, GCKR has emerged as a prominent target for the treatment of T2DM. The previous strategy indirectly enhances activity and broadens the range of GCK via the alleviation of GCKR -mediated repression. Recent studies have revealed that increased GCKR expression can correct impaired glucose metabolism, and GCKR overexpression in T2DM mice has shown long-term beneficial effects on glucose metabolism [38]. Indeed, genetic evidence suggests that the impaired interaction of the GCK- GCKR complex may increase the risk of liver and cardiovascular diseases; therefore, the development of future GCK activators should avoid interfering with the GCK- GCKR interaction and should instead activate liver-specific GCK [39,40].

In the present gene and protein expression analysis of candidate proteins for hypoglycemic activity, we focused on the hub protein/gene of GCK/*Gck*, which had 2.86/2.18-fold upregulated expression in the GF5000-treated group compared to that in the DC group. There is a negative correlation between the mRNA or protein expression of GCK and blood sugar levels in T2DM [41]; therefore, we validated the stimulating effect of GF5000 on *GCK* expression using *GCK* knockdown in HepG2 cells. It was interesting that a concentration of 20 mg/mL of GF5000 may increase the mRNA expression of *GCK*.

Therefore, it is reasonable to conclude, through comprehensive analyses of transcriptomic/proteomic datasets and network pharmacology, that GCK is pivotal for the hypoglycemic activity of GF5000 and that the activation of GCK causes the acceleration of glycolysis and promotes the use of the body, thus achieving the purpose of lowering blood glucose. In addition, the amino acid metabolic pathway that is enriched by GF5000 also undergoes changes in its BCAA and AAA contents, which may lead to a hypoglycemic effect by improving insulin resistance through the amino acid-mTORC1-insulin signaling pathway [42].

However, the entire hypoglycemic mechanism remains unclear. If the metabolome can be combined, a network relationship between genes, proteins, and metabolites can be constructed for further research and analysis. With this, the hypoglycemic mechanism of *G. frondosa* GF5000 will be further uncovered.

## 5. Conclusions

Through a comprehensive analysis of transcriptome/proteome datasets and network pharmacology, followed by cell-based experiments, it was demonstrated that GCK is crucial in the hypoglycemic activity of GF5000 and that amino acid metabolism alteration may be the main pathway by which insulin resistance is improved. These results provide theoretical evidence that *G. frondosa* GF5000 is a potential active nutritional ingredient for the prevention and treatment of T2DM.

## Figures and Tables

**Table 1:** Top 20 small molecules predicted by cMAP analysis (score > 0.7).

Rank	Perturbagen	Score	Dose	Cell	Up	Down	ATC
1	Enalapril	0.865	8 µM	MCF7	0.233	−0.216	C
2	Tribenoside	0.857	8 µM	HL60	0.303	−0.142	C
3	Metformin	0.854	24 µM	HL60	0.204	−0.24	A
4	Biotin	0.832	16 µM	PC3	0.332	−0.1	A
5	Methotrexate	0.827	9 µM	HL60	0.275	−0.154	L
6	Furosemide	0.797	12 µM	HL60	0.24	−0.173	C
7	Chenodeoxycholic acid	0.796	10 µM	MCF7	0.233	−0.18	A
8	Pindolol	0.792	16 µM	PC3	0.249	−0.162	C
9	Lomustine	0.791	100 µM	PC3	0.232	−0.179	L
10	Lomustine	0.789	100 µM	MCF7	0.249	−0.16	L
11	Mesalazine	0.787	26 µM	MCF7	0.245	−0.164	A
12	Cyclopenthiazide	0.782	11 µM	HL60	0.194	−0.212	C
13	Rosiglitazone	0.779	10 µM	MCF7	0.235	−0.169	A
14	Troglitazone	0.777	10 µM	PC3	0.278	−0.125	A
15	Nifedipine	0.772	12 µM	PC3	0.144	−0.257	C
16	Ciclosporin	0.768	3 µM	PC3	0.204	−0.194	L
17	Vinblastine	0.762	100 nM	MCF7	0.197	−0.199	L
18	Rescinnamine	0.755	6 µM	HL60	0.225	−0.166	C
19	Sirolimus	0.755	100 nM	HL60	0.209	−0.182	L
20	Rilmenidine	0.751	8 µM	PC3	0.197	−0.193	C

The ATC (Anatomical Therapeutic Chemical) classification has a 7-bit code, the first of which is the first letter, showing the anatomical classification. A: alimentary tract and metabolism; C: cardiovascular system; L: antineoplastic and immunomodulating agents.

**Table 2:** Expression of two DEGs and DEPs with the same trend in proteomic and transcriptomic analyses in the GF5000-VS-DC comparison.

No.	Protein/Gene ID	Protein/Gene Name	Protein Ratio (GF5000/DC)		Gene Ratio (GF5000/DC)	
			Mean Ratio	Q-Value	Log2 Ratio	Probability
1	O08651/58835	D-3-phosphoglycerate dehydrogenase/*Phgdh*	3.38	0.002|0.191|0.004	3.19	0.898
2	P12785/50671	Fatty acid synthase/*Fasn*	2.77	0.002|0.004|0.002	2.43	0.880
3	Q64611/60356	Cysteine sulfinic acid decarboxylase/*Csad*	1.85	0.002|0.009|0.008	2.37	0.917
4	P17712/24385	Glucokinase/*Gck*	2.86	0.002|0.007|0.002	2.18	0.855
5	P55053/140868	Fatty acid-binding protein/*Fabp5*	1.73	0.002|0.659|0.002	1.99	0.880
6	P14141/54232	Carbonic anhydrase 3/*Car3*	1.92	0.002|0.004|0.002	1.84	0.901
7	P16638/24159	ATP-citrate synthase/*Acly*	1.9	0.002|0.081|0.002	1.47	0.830
8	P14046/297568	alpha-1-inhibitor III/*LOC297568*	1.61	0.002|0.004|0.002	1.32	0.865
9	B1WBN9/24651	pyruvate kinase PKLR/*Pklr*	2.05	0.002|0.013|0.002	1.17	0.841
10	P50137/64524	Transketolase/*Tkt*	1.47	0.002|0.841|0.002	1.06	0.824
11	A0A096MJY6/288333	1,4-alpha-glucan-branching enzyme 1/*Gbe1*	1.58	0.002|0.004|0.002	1.05	0.811
12	P04182/64313	Ornithine aminotransferase/*Oat*	0.43	0.282|0.607|0.414	−1.58	0.876
13	P05182/25086	Cytochrome P450 2E1/*Cyp2e1*	0.47	0.003|0.091|0.004	−1.87	0.904
14	P25409/81670	Alanine aminotransferase 1/*Gpt*	0.44	0.003|0.049|0.002	−1.23	0.850
15	P32755/29531	4-hydroxyphenylpyruvate dioxygenase/*Hpd*	0.61	0.002|0.004|0.002	−1.24	0.856
16	Q9Z2M4/64461	Peroxisomal 2,4-dienoyl-CoA reductase/*Decr2*	0.58	0.018|0.004|0.072	−1.06	0.818
17	Q63581/24903	T-kininogen 1/*Kng1*	0.79	0.033|0.831|0.012	−1.08	0.836
18	Q4V8F9/313200	Hydroxysteroid dehydrogenase-like protein 2/*Hsdl2*	0.43	0.002|0.126|0.009	−1.11	0.804
19	G3V734/117543	2,4-dienoyl CoA reductase 1/*Decr1*	0.61	0.004|0.017|0.058	−1.19	0.844
20	Q6SKG1/24763	Acyl-coenzyme A synthetase ACSM3, mitochondrial/*Acsm3*	0.55	0.002|0.206|0.004	−1.32	0.833
21	Q62651/64526	Delta(3,5)-Delta(2,4)-dienoyl-CoA isomerase, mitochondrial/*Ech1*	0.73	0.002|0.795|0.002	−1.43	0.869
22	P80299/65030	Bifunctional epoxide hydrolase 2/*Ephx2*	0.57	0.002|0.024|0.004	−1.93	0.892
23	Q68G41/29740	Enoyl-CoA delta isomerase 1, mitochondrial/*Eci1*	0.55	0.002|0.004|0.002	−1.94	0.902

**Table 3:** The thirteen common genes selected from supplemental Tables S3 and S4.

Number	Protein Description	Gene Symbol (Rat)/(Human)	Protein Ratio (GF5000/DC)		Gene Ratio (GF5000/DC)	
			Mean Ratio	Q-Value	Log2 Ratio	Probability
1	Bile salt export Pump	*Abcb4*/*ABCB11*	/		−2.20	0.889129025
2	Acetyl-CoA Carboxylase 1	*Acaca*/*ACACB*	2.23	0.002|0.135|0.002	−1.94	0.902120404
3	Aldehyde oxidase	*Aox1*/*AOX1*	1.15	0.172|0.018|0.002	/	
4	Apolipoprotein A-I	*Apoa1*/*APOA1*	0.57	0.002|0.021|0.002	/	
5	Glucokinase	*Gck*/*GCK*	2.86	0.002|0.007|0.002	2.18	0.855341834
6	Glucokinase regulatory protein	*Gckr*/*GCKR*	1.39	0.004|0.770|0.037	/	
7	Glutamine synthetase	*Glul*/*GLUL*	/		−1.26	0.853012511
8	Acyl-CoA desaturase 1	*Scd*/*SCD*	/		2.72	0.94223519
9	Histone cluster 1 H1 family member c	*Hist1h1c*/*HIST1H1B*	1.27	0.002|0.004|0.002	/	
10	Pre-mRNA-processing factor 6	*Prpf6*/*PRPF6*	1.90	0.008|0.004|0.243	/	
11	Serpin H1	*Serpinh1*/*SERPINH1*	0.56	0.003|0.781|0.020	/	
12	SH3 and cysteine rich domain 3	*Stac3*/*STAC3*	/		3.09	0.94514084
13	Cytochrome P450 3A9	*Cyp3a9*/*TBXAS1*	/		−3.15	0.936207766

**Table 4:** Different types of interactions associated with the AGs.

Type of Interaction	Interaction (%)
Predicted	63.97%
Co-expression	22.02%
Physical Interactions	12.81%
Co-localization	1.19%

**Table 5:** AGs with the highest centralities and their interacting genes and networks based on the GeneMANIA network map.

Gene	Protein Description	Degree Centrality	Interacting Gene	Interaction Type of Networks *
*Apoa1*	Apolipoprotein A-I	13	*Gckr, Cyp3a9, Pon1, Apoc1, Txndc2, Spef2, Qdpr, C1qa, Pde4b,* *Tomm20, Hba-a1, Apof*	1,3,4
*Gckr*	Glucokinase regulatory protein	10	*Stac3, Qdpr, Scd, Apoa1, Cyp3a9, Gck, Glud1*	1,2,3
*Gck*	Glucokinase	9	*Ins2, Gckr, Pfkfb1, Glud1, Pfkfb3, Pfkfb2,*	1,2,3
*Cyp3a9*	Cytochrome P450 3A9	6	*Glud1, Pon1, Aox1, Glul, Gckr, Apoa1*	1,4
*Scd*	Acyl-CoA desaturase 1	4	*Stac3, Gckr, Pfkfb1, Cyb5a*	1,3
*Glul*	Glutamine synthetase	4	*Cyp3a9, Htr2a, Glud1, Nudt18*	1,3
*Aox1*	Aldehyde oxidase 1	2	*Cyp3a9, Cyb5a*	1
*Serpinh1*	Serpin H1	2	*Nudt18, Qdpr*	1
*Stac3*	SH3 and cysteine rich domain 3	2	*Scd, Gckr*	1
*Prpf6*	Pre-mRNA-processing factor 6	2	*Araf, Rnps1*	3
*Hist1h1c*	Histone cluster 1 H1 family member c	1	*Nudt18*	3

* 1—Co-expression; 2—physical interactions; 3—predicted; 4—co-localization.

## Data Availability

The authors declare that all of the data and materials used in this study are available within this article. All of the data generated or analyzed in this study can be obtained from the authors upon reasonable request.

## Supplementary Materials

The following supporting information can be downloaded at https://www.mdpi.com/article/10.3390/nu17060964/s1: Table S1: T2DM targets; Table S2: hprdPPI; Table S3: T2DM drug targets and their neighbor nodes; Table S4: DEGs and DEPs (human).

## Abbreviations

The following abbreviations are used in this manuscript:

AMPK-α	Activated protein kinase-α
APOA-1	Apolipoprotein A-I
BMI	Body mass index
cMAP	Connectivity MAP analysis
GCK	Glucokinase
GCKR	Glucokinase regulatory protein
GLUT4	Glucose transporter 4
GO	Gene ontology
GSEA	Gene-set enrichment analysis
GSK-3	Glycogen synthase kinase 3
HbA1c	Hemoglobin A1c
IR	Insulin receptor
IRS1	Insulin receptor substrate 1
KEGG	Kyoto Encyclopedia of Genes and Genomes
PKB	Protein kinase B
PI3K	Phosphatidylinositol-3-kinase
T2DM	Type 2 diabetes mellitus
TG	Triglyceride
STZ	Streptozotocin
